# Dataset exploring organizational culture of K-12 schools

**DOI:** 10.1016/j.dib.2022.108179

**Published:** 2022-04-12

**Authors:** Jacqueline Kareem, Harold Andrew Patrick, Veerta Tantia, Sharon Valarmathi B

**Affiliations:** a School of Education, Christ University, Bangalore, India; b CMS Business School, Jain University, Bangalore, India; c School of Commerce Finance and Accountancy, Christ University, Bangalore, India

**Keywords:** Organizational culture, OCTAPACE, School culture

## Abstract

Culture can be understood as an explicit social product arising from social interaction as an intentional or unintentional consequence of behavior. Educational Institutions culture differs from other organizational cultures as it impacts teachers' performance and students' learning. In this survey the definition of organizational culture used is given by Schein, “The deeper level of basic assumptions and beliefs that are, learned responses to the group's problems of survival in its external environment and its problems of internal integration; are shared by members of an organization; that operate unconsciously; and that define in a basic ‘taken -for-granted’ fashion in an organization's view of itself and its environment” [1]. The data contains 1158 cases collected from K-12 School teachers on their perception of values and beliefs of their organizational culture using the OCTAPACE scale. Convenience sampling is used to obtain the data from teachers. The questionnaire was administered personally to teachers from sixty-five Private aided, Private unaided and Government schools. The eight dimensions measuring values and beliefs of Educational Institutions organizational culture are Pro-action, Authenticity, Openness, Collaboration, Experimenting, Trust, Confrontation and Autonomy. Descriptive statistics are computed from the dataset. The dataset can be used by researchers for meta analysis on organizational culture and school management can explore in depth the need for an organizational culture of autonomy, experimenting, collaboration and openness among teachers.

## Specifications Table

**Table:** 

Subject	Social science - Education
Specific subject area	Educational Organizational Culture
Type of data	Tables
How the data were acquired	The data was acquired through a self-reported survey tool. The tool has two parts, namely the demographic profile of the respondent and the OCTAPACE scale measuring the values and beliefs of organizational culture.
Data format	Raw Analyzed
Description of data collection	Data was collected from teachers personally. They were briefed about the survey. The survey was a self-reported questionnaire. Data was collected from school teachers who consented to answering the survey. Data was screened for missing values. Data that passed the normality test was considered for analysis. A final sample of 1158 responses was found to be valid. The questionnaire is provided in the Mendeley dataset.
Data source location	Data was collected from teachers of sixty-five schools, from the city of Bangalore, India.
Data accessibility	Data is hosted on: Repository name: Mendeley Data Data identification number: DOI:10.17632/9z7xzvtsd2.3 Direct URL to data: https://data.mendeley.com/datasets/9z7xzvtsd2/3

## Value of the Data

• The data can be used by Management of schools to properly make decisions that in the long-run would lead to create a conducive environment for teachers and students.
• The data can be used to enlighten principals and other authorities to know the importance of organizational culture and how it can be beneficial to the overall performance of the educational institutions.
• The data will help in the training of teachers and administrators for creating an effective learning environment.
• It will help researchers to conduct meta- analysis studies on organizational culture and perform confirmatory factor analysis.
• The data set will be useful for providing hands-on training in quantitative statistical packages.

## 1 Data Description

Table 1 describes the sample distribution based on demographics. The skewness values of openness (−0.603), confrontation (−0.345), trust (−0.228), authenticity (−0.463), pro-action (−0.678), autonomy (−0.390), collaboration (−0.918) and experimenting (−0.562) is less than +/−1.96, hence the data does not deviate from normality. Table 2 shows the correlation values for the dimensions of organizational culture. Table 3, Table 4, Table 5, Table 6, Table 7, Table 8, Table 9, Table 10, Table 11, Table 12 displays the means and standard deviation of the eight dimensions of organizational culture across the demographic variables. In Mendeley database [2] the following are provided: (1) The survey questionnaire (2) Organizational culture data-1158.sav (3) Organizational culture dataset-1158.xls.

**Table 1:** Sample profile distribution.

Demographics	Value	Count	Percent
School Board	State	931	80.4%
	ICSE	179	15.5%
	CBSE	48	4.1%
School Management	Government	355	30.7%
	Aided	374	32.3%
	Unaided/Private	429	37.0%
Gender	Female	961	83.0%
	Male	197	17.0%
Marital Status	Married	868	75.0%
	Unmarried	290	25.0%
Age	<20 Yrs	6	0.5%
	21–25 Yrs	110	9.5%
	26–30 Yrs	276	23.8%
	31–35 Yrs	145	12.5%
	36–40 Yrs	137	11.8%
	41–45 Yrs	187	16.1%
	>46 Yrs	297	25.6%
Educational Qualification	Diploma	143	12.3%
	Graduation	519	44.8%
	Post Graduation	371	32.0%
	Others	125	10.8%
Years of Experience	<2 Yrs	121	10.4%
	2–5 Yrs	271	23.4%
	6–10 Yrs	254	21.9%
	11–20 Yrs	216	18.7%
	>20 Yrs	296	25.6%
Teaching subject	Languages	373	32.2%
	Humanities(Social science)	267	23.1%
	Sciences (incl. Math)	330	28.5%
	Extra curricular/others	188	16.2%
Monthly income	<Rs. 5000	46	4.0%
	Rs. 5000–15,000	536	46.3%
	Rs. 15,001–30,000	536	46.3%
	>Rs. 30,000	40	3.5%
School Level	Primary	319	27.5%
	Middle	339	29.3%
	High/Secondary	354	30.6%
	Higher Secondary	146	12.6%

**Table 2:** Karl Pearson's coefficient of correlations.

	OP	CF	TR	AC	PA	AT	CL	EX
Openness(OP)	--							
Confrontation(CF)	.439^⁎⁎^	--						
Trust(TR)	.569^⁎⁎^	.530^⁎⁎^	--					
Authenticity(AC)	.335^⁎⁎^	.451^⁎⁎^	.262^⁎⁎^	--				
Pro-Action(PA)	.626^⁎⁎^	.436^⁎⁎^	.496^⁎⁎^	.481^⁎⁎^	--			
Autonomy(AT)	.378^⁎⁎^	.574^⁎⁎^	.404^⁎⁎^	.410^⁎⁎^	.461^⁎⁎^	--		
Collaboration(CL)	.549^⁎⁎^	.617^⁎⁎^	.497^⁎⁎^	.473^⁎⁎^	.578^⁎⁎^	.550^⁎⁎^	--	
Experimenting(EX)	.421^⁎⁎^	.637^⁎⁎^	.452^⁎⁎^	.546^⁎⁎^	.563^⁎⁎^	.547^⁎⁎^	.687^⁎⁎^	--

**Table 3:** Means of organizational culture dimensions across Type of Board categories.

Type of Board		N	Mean	Std. Deviation
State	Openness	931	12.43	2.645
	Confrontation	931	11.47	2.252
	Trust	931	11.58	2.254
	Authenticity	931	8.38	1.985
	Pro-Action	931	12.53	2.373
	Autonomy	931	8.64	1.916
	Collaboration	931	12.31	2.539
	Experimenting	931	12.16	2.969
			
ICSE	Openness	179	11.95	2.623
	Confrontation	179	12.06	2.388
	Trust	179	11.94	2.292
	Authenticity	179	7.98	1.820
	Pro-Action	179	12.22	1.987
	Autonomy	179	8.80	1.917
	Collaboration	179	12.46	2.234
	Experimenting	179	12.64	2.338
			
CBSE	Openness	48	13.13	2.275
	Confrontation	48	12.38	2.160
	Trust	48	12.46	2.414
	Authenticity	48	8.27	1.484
	Pro-Action	48	12.27	2.081
	Autonomy	48	9.04	1.701
	Collaboration	48	12.37	2.429
	Experimenting	48	12.42	2.350

**Table 4:** Means of organizational culture dimensions across Type of School categories.

Type of School		N	Mean	Std. Deviation
Government	Openness	355	12.85	1.758
	Confrontation	355	11.69	2.126
	Trust	355	11.62	1.735
	Authenticity	355	8.98	1.835
	Pro-Action	355	12.93	1.784
	Autonomy	355	8.76	1.512
	Collaboration	355	12.70	2.095
	Experimenting	355	12.76	3.001
				
Aided	Openness	374	12.33	2.987
	Confrontation	374	11.14	2.155
	Trust	374	11.42	2.428
	Authenticity	374	8.15	1.895
	Pro-Action	374	12.31	2.441
	Autonomy	374	8.58	2.058
	Collaboration	374	12.28	2.282
	Experimenting	374	11.72	2.710
				
Unaided/Private	Openness	429	12.05	2.856
	Confrontation	429	11.92	2.453
	Trust	429	11.95	2.495
	Authenticity	429	7.91	1.943
	Pro-Action	429	12.25	2.516
	Autonomy	429	8.71	2.062
	Collaboration	429	12.09	2.899
	Experimenting	429	12.28	2.794

**Table 5:** Means of organizational culture dimensions across Gender categories.

Gender		N	Mean	Std. Deviation
Female	Openness	961	12.42	2.621
	Confrontation	961	11.83	2.174
	Trust	961	11.78	2.324
	Authenticity	961	8.50	1.878
	Pro-Action	961	12.62	2.255
	Autonomy	961	8.87	1.779
	Collaboration	961	12.59	2.368
	Experimenting	961	12.62	2.681
Male	Openness	197	12.22	2.707
	Confrontation	197	10.45	2.461
	Trust	197	11.15	1.936
	Authenticity	197	7.42	2.028
	Pro-Action	197	11.77	2.432
	Autonomy	197	7.76	2.227
	Collaboration	197	11.11	2.699
	Experimenting	197	10.42	3.005

**Table 6:** Means of organizational culture dimensions across Marital Status categories.

Marital Status		N	Mean	Std. Deviation
Married	Openness	868	12.49	2.812
	Confrontation	868	11.44	2.341
	Trust	868	11.69	2.425
	Authenticity	868	8.16	1.891
	Pro-Action	868	12.38	2.356
	Autonomy	868	8.66	2.041
	Collaboration	868	12.24	2.543
	Experimenting	868	11.97	2.827
				
Unmarried	Openness	290	12.06	1.987
	Confrontation	290	12.06	2.037
	Trust	290	11.63	1.750
	Authenticity	290	8.77	2.039
	Pro-Action	290	12.77	2.134
	Autonomy	290	8.77	1.443
	Collaboration	290	12.63	2.298
	Experimenting	290	13.07	2.806

**Table 7:** Means of organizational culture dimensions across Age categories.

Age		N	Mean	Std. Deviation
<20 Yrs	Openness	6	11.83	2.858
	Confrontation	6	11.17	.983
	Trust	6	12.17	1.722
	Authenticity	6	8.17	.408
	Pro-Action	6	10.00	1.095
	Autonomy	6	7.67	1.211
	Collaboration	6	11.50	2.168
	Experimenting	6	12.00	2.191
21–25 Yrs	Openness	110	11.75	2.194
	Confrontation	110	11.75	2.043
	Trust	110	11.63	1.876
	Authenticity	110	8.31	2.149
	Pro-Action	110	12.68	2.534
	Autonomy	110	8.70	1.923
	Collaboration	110	12.03	2.791
	Experimenting	110	12.20	3.298
				
26–30 Yrs	Openness	276	12.19	2.268
	Confrontation	276	11.80	2.209
	Trust	276	11.49	1.875
	Authenticity	276	8.68	1.952
	Pro-Action	276	12.52	2.198
	Autonomy	276	8.72	1.675
	Collaboration	276	12.46	2.475
	Experimenting	276	12.82	2.823
31–35 Yrs	Openness	145	12.57	2.783
	Confrontation	145	11.37	2.516
	Trust	145	11.66	2.247
	Authenticity	145	8.22	1.884
	Pro-Action	145	12.17	2.354
	Autonomy	145	8.23	2.000
	Collaboration	145	11.86	2.483
	Experimenting	145	11.17	2.843
36–40 Yrs	Openness	137	12.50	2.983
	Confrontation	137	10.98	2.608
	Trust	137	11.74	2.317
	Authenticity	137	7.97	2.047
	Pro-Action	137	12.38	2.383
	Autonomy	137	8.55	2.128
	Collaboration	137	11.77	3.056
	Experimenting	137	11.94	2.884
41–45 Yrs	Openness	187	12.89	2.583
	Confrontation	187	11.49	2.218
	Trust	187	11.99	2.305
	Authenticity	187	8.12	2.029
	Pro-Action	187	12.71	1.891
	Autonomy	187	8.61	1.850
	Collaboration	187	12.64	2.254
	Experimenting	187	11.98	2.902
>46 Yrs	Openness	297	12.35	2.835
	Confrontation	297	11.82	2.165
	Trust	297	11.64	2.692
	Authenticity	297	8.31	1.765
	Pro-Action	297	12.46	2.482
	Autonomy	297	8.99	1.959
	Collaboration	297	12.66	2.144
	Experimenting	297	12.56	2.524

**Table 8:** Means of organizational culture dimensions across Educational Qualification categories.

Educational Qualification		N	Mean	Std. Deviation
Diploma	Openness	143	12.40	2.784
	Confrontation	143	11.71	1.920
	Trust	143	11.55	2.860
	Authenticity	143	8.14	1.568
	Pro-Action	143	12.35	2.386
	Autonomy	143	9.06	1.924
	Collaboration	143	12.50	1.776
	Experimenting	143	12.53	2.343
				
Graduation	Openness	519	12.74	2.426
	Confrontation	519	11.60	2.374
	Trust	519	11.85	2.096
	Authenticity	519	8.51	2.029
	Pro-Action	519	12.66	2.175
	Autonomy	519	8.58	1.931
	Collaboration	519	12.34	2.632
	Experimenting	519	12.20	2.969
				
Post Graduation	Openness	371	12.23	2.519
	Confrontation	371	11.57	2.241
	Trust	371	11.68	2.118
	Authenticity	371	8.16	1.911
	Pro-Action	371	12.35	2.296
	Autonomy	371	8.66	1.853
	Collaboration	371	12.27	2.502
	Experimenting	371	12.18	2.995
				
Others	Openness	125	11.37	3.286
	Confrontation	125	11.54	2.425
	Trust	125	11.09	2.584
	Authenticity	125	8.18	2.036
	Pro-Action	125	12.21	2.719
	Autonomy	125	8.74	1.926
	Collaboration	125	12.31	2.554
	Experimenting	125	12.30	2.518

**Table 9:** Means of organizational culture dimensions across Years of Experience categories.

Years of Experience		N	Mean	Std. Deviation
<2 Yrs	Openness	121	12.05	2.493
	Confrontation	121	12.14	2.018
	Trust	121	11.88	2.274
	Authenticity	121	8.90	1.781
	Pro-Action	121	12.99	2.304
	Autonomy	121	8.93	1.694
	Collaboration	121	12.78	2.174
	Experimenting	121	12.97	2.633
				
2–5 Yrs	Openness	271	12.09	2.413
	Confrontation	271	11.73	2.443
	Trust	271	11.66	1.849
	Authenticity	271	8.34	2.173
	Pro-Action	271	12.43	2.294
	Autonomy	271	8.54	1.827
	Collaboration	271	12.13	2.758
	Experimenting	271	12.32	3.176
				
6–10 Yrs	Openness	254	12.74	2.619
	Confrontation	254	11.06	2.375
	Trust	254	11.46	2.059
	Authenticity	254	7.81	1.909
	Pro-Action	254	12.15	2.316
	Autonomy	254	8.13	2.022
	Collaboration	254	11.74	2.752
	Experimenting	254	11.19	3.062
				
11–20 Yrs	Openness	216	12.35	2.748
	Confrontation	216	11.51	2.237
	Trust	216	11.94	2.488
	Authenticity	216	8.23	1.805
	Pro-Action	216	12.49	2.212
	Autonomy	216	8.86	1.866
	Collaboration	216	12.62	2.408
	Experimenting	216	12.24	2.609
				
>20 Yrs	Openness	296	12.51	2.785
	Confrontation	296	11.77	2.103
	Trust	296	11.60	2.607
	Authenticity	296	8.56	1.822
	Pro-Action	296	12.58	2.352
	Autonomy	296	9.06	1.883
	Collaboration	296	12.64	2.027
	Experimenting	296	12.79	2.360

**Table 10:** Means of organizational culture dimensions across Teaching Subjects categories.

Teaching Subjects		N	Mean	Std. Deviation
Languages	Openness	373	12.50	2.674
	Confrontation	373	11.66	2.230
	Trust	373	11.84	2.287
	Authenticity	373	8.18	1.888
	Pro-Action	373	12.54	2.333
	Autonomy	373	8.76	1.922
	Collaboration	373	12.43	2.588
	Experimenting	373	12.37	2.923
				
Humanities(Social science)	Openness	267	12.22	2.684
	Confrontation	267	11.54	2.319
	Trust	267	11.78	2.229
	Authenticity	267	8.29	1.971
	Pro-Action	267	12.37	2.296
	Autonomy	267	8.46	1.916
	Collaboration	267	11.99	2.563
	Experimenting	267	11.97	2.915
				
Sciences (incl. Math)	Openness	330	12.35	2.627
	Confrontation	330	11.56	2.286
	Trust	330	11.58	2.336
	Authenticity	330	8.34	1.996
	Pro-Action	330	12.50	2.411
	Autonomy	330	8.66	1.881
	Collaboration	330	12.38	2.517
	Experimenting	330	12.31	2.932
Extra curricular/others	Openness	188	12.46	2.511
	Confrontation	188	11.60	2.348
	Trust	188	11.36	2.181
	Authenticity	188	8.57	1.927
	Pro-Action	188	12.46	2.090
	Autonomy	188	8.88	1.903
	Collaboration	188	12.55	2.067
	Experimenting	188	12.28	2.504

**Table 11:** Means of organizational culture dimensions across Monthly Income categories.

Monthly Income		N	Mean	Std. Deviation
< Rs. 5000	Openness	46	10.02	3.343
	Confrontation	46	9.61	2.720
	Trust	46	10.65	2.368
	Authenticity	46	5.87	1.962
	Pro-Action	46	10.04	2.996
	Autonomy	46	7.13	2.306
	Collaboration	46	8.89	3.677
	Experimenting	46	9.37	3.172
				
Rs. 5000–15,000	Openness	536	12.58	2.690
	Confrontation	536	11.60	2.355
	Trust	536	11.84	2.274
	Authenticity	536	8.13	1.796
	Pro-Action	536	12.65	2.313
	Autonomy	536	8.68	1.934
	Collaboration	536	12.30	2.481
	Experimenting	536	12.22	2.806
				
Rs. 15,001–30,000	Openness	536	12.34	2.432
	Confrontation	536	11.77	2.087
	Trust	536	11.54	2.238
	Authenticity	536	8.70	1.951
	Pro-Action	536	12.51	2.143
	Autonomy	536	8.84	1.771
	Collaboration	536	12.69	2.175
	Experimenting	536	12.50	2.761
				
> Rs. 30,000	Openness	40	13.13	2.267
	Confrontation	40	11.45	2.298
	Trust	40	12.33	2.258
	Authenticity	40	8.60	1.355
	Pro-Action	40	12.58	1.960
	Autonomy	40	8.40	2.073
	Collaboration	40	12.00	1.725
	Experimenting	40	12.42	2.800

**Table 12:** Means of organizational culture dimensions across School Level categories.

School Level		N	Mean	Std. Deviation
Primary	Openness	319	12.13	2.927
	Confrontation	319	11.89	2.153
	Trust	319	11.48	2.695
	Authenticity	319	8.32	1.790
	Pro-Action	319	12.55	2.345
	Autonomy	319	8.94	1.890
	Collaboration	319	12.57	2.361
	Experimenting	319	12.68	2.385
				
Middle	Openness	339	12.12	2.850
	Confrontation	339	11.36	2.334
	Trust	339	11.71	2.247
	Authenticity	339	8.05	2.023
	Pro-Action	339	12.18	2.576
	Autonomy	339	8.65	2.037
	Collaboration	339	12.14	2.659
	Experimenting	339	12.04	3.020
				
High/Secondary	Openness	354	12.73	2.253
	Confrontation	354	11.62	2.348
	Trust	354	11.77	2.041
	Authenticity	354	8.50	1.947
	Pro-Action	354	12.55	2.053
	Autonomy	354	8.67	1.807
	Collaboration	354	12.32	2.528
	Experimenting	354	12.16	2.767
				
Higher Secondary	Openness	146	12.72	2.152
	Confrontation	146	11.44	2.228
	Trust	146	11.78	1.825
	Authenticity	146	8.50	2.035
	Pro-Action	146	12.84	2.081
	Autonomy	146	8.22	1.802
	Collaboration	146	12.29	2.226
	Experimenting	146	11.97	3.508

## 2 Experimental Design, Materials and Methods

A survey was conducted among K-12 School teachers of private and government institutions to construct this dataset. We identified sixty-five schools through the convenience sample method. A total of 1158 teachers' responses are considered in this dataset. The questionnaire is self administered by the school teachers. To maintain confidentiality, the identity of the teachers and the school names are not disclosed in the survey. The data were analyzed using SPSS software. Descriptive statistics was computed. A standardized tool measuring organizational culture - OCTAPACE scale [3] is used in this survey.

The survey tool collected data on ten demographics of the teachers namely; Type of Board(Syllabus), Type of School(Management), Gender, Marital status, Age, Educational qualifications, Years of teaching experience, primary Teaching subject and Level of School. To measure the value and beliefs of organizational culture the OCTPACE scale was used. The OCTAPACE scale is primarily a forty item instrument that gives the profile of institutions ' ethos in eight dimensions, the dataset has data for thirty items more relevant to Indian schools. The eight dimensions of the OCTAPACE scale are: Pro-action, Authenticity, Openness, Collaboration, Experimenting, Trust, Confrontation and Autonomy. The scale contains 2 parts, Part-1, 22 items for the 8 dimensions is stated and the respondent is required to check on a 4-point scale how much each item is valued in their organization. The rating is 1=very low value, 2=rather low value, 3=fairly high value, and 4=highly valued. Part-2 contains 8 items on beliefs for the 8 dimensions stated and the respondent is required to check on a 4-point scale how widely each item is shared in their organization. The rating is 1=only a few or none share this belief, 2=only some share this belief, 3=fairly widely shared belief, and 4=very widely shared belief.

## Ethics Statements

The authors: Dr Jacqueline Kareem, Prof Harold Andrew Patrick, Dr Veerta Tantia and Dr Sharon Valarmathi hereby declare that this dataset has not been previously published elsewhere, nor is this dataset considered for publication elsewhere. Before collecting data from the respondents, a clear explanation was provided to the respondent of the research objective. The respondent's consent was duly obtained for publication of the data. We also received approval from the Research Ethics Committee (REC) of CHRIST (Deemed to be University), Bangalore (CU: RCEC/00217/08/21) After reviewing the objective, methodology of the survey, informed consent and the questionnaire, the Committee approved the survey. Respondents were communicated that the survey is used exclusively for academic purposes.

## CRediT authorship contribution statement

**Jacqueline Kareem:** Conceptualization, Methodology, Investigation, Formal analysis. **Harold Andrew Patrick:** Conceptualization, Methodology, Investigation, Supervision. **Veerta Tantia:** Writing – original draft, Investigation. **Sharon Valarmathi B:** Investigation, Writing – review & editing.

## Declaration of Competing Interest

We as authors declare that we have no known competing financial interests or personal relationships, which have or could be perceived to have influenced our work reported in this article. The research is a non-funded survey.

## Data Availability

Organizational Culture (Original data) (Mendeley Data).
